# In Flanders Fields: John McCrae as Physician, Poet, Soldier

**DOI:** 10.14797/mdcvj.1283

**Published:** 2023-11-16

**Authors:** James C. Young

**Affiliations:** 1 Department of Anesthesiology, Walter Reed National Military Medical Center, Bethesda, Maryland, US

## Abstract

John McCrae (1872–1918) was a Canadian physician, poet, and soldier who fought and died in the First World War. He penned perhaps his most memorable and lasting poem, “In Flanders Fields,” shortly after the death of a comrade at the Second Battle of Ypres in 1915. The poem gained almost instant popularity, being used for recruiting efforts and victory bond sales throughout the remainder of the war, and solidified forever the symbol of the poppy as a memorial token for the service members who had perished. His death towards the end of the war, like that of so many others in the perilous years between 1914 and 1918, cut short the trajectory of what had already amounted to a brilliant career. As a close friend of such titans of medicine as William Osler and Harvey Cushing, as well as acquainted with the likes of Rudyard Kipling, it is not difficult to imagine the impact that his passing had upon the future of medicine and literature.

## In Flanders Fields

In Flanders fields the poppies blow
Between the crosses, row on row,
    That mark our place; and in the sky
    The larks, still bravely singing, fly
Scarce heard amid the guns below.

We are the Dead. Short days ago
We lived, felt dawn, saw sunset glow,
    Loved and were loved, and now we lie,
        In Flanders fields.

Take up our quarrel with the foe:
To you from failing hands we throw
    The torch; be yours to hold it high.
    If ye break faith with us who die
We shall not sleep, though poppies grow
        In Flanders fields.

John McCrae
*In Flanders Fields and Other Poems* (New York: GP Putnam’s Sons 1919)

This poem is in the public domain.

**Image 1:**
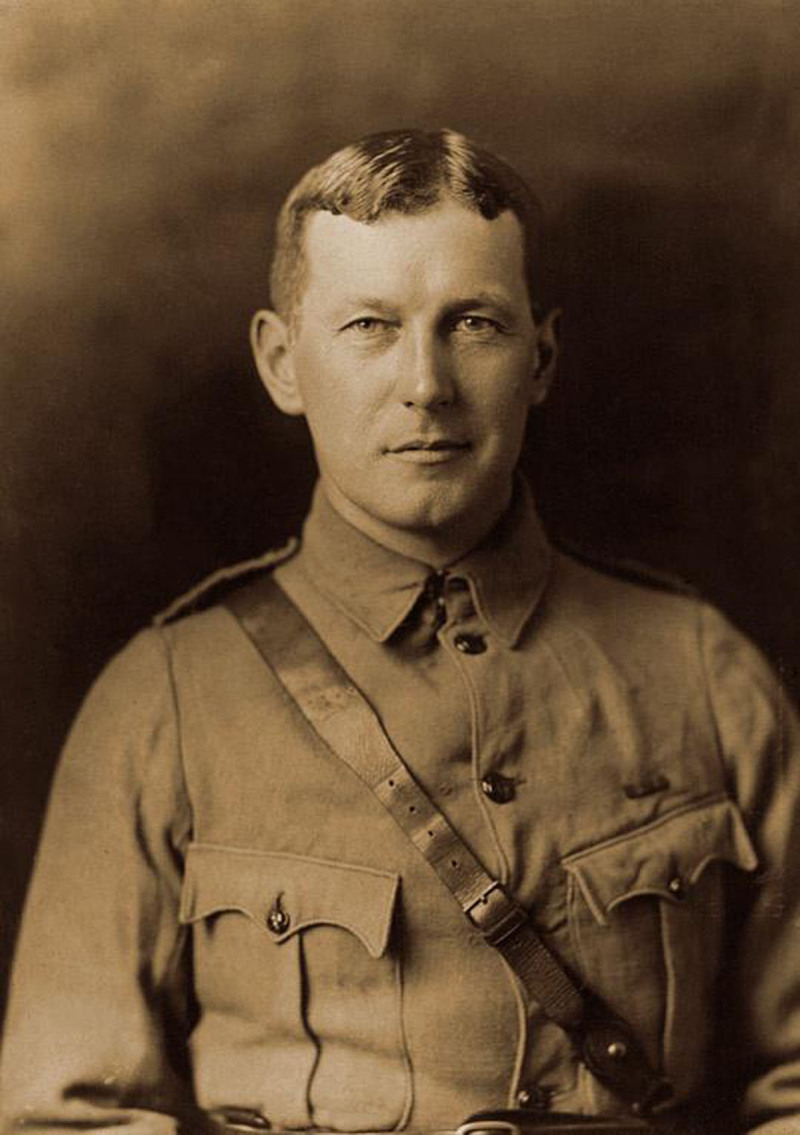
John McCrae in uniform circa 1914. Source: William Notman and Son – Guelph Museums, Reference No. M968.354

**Image 2:**
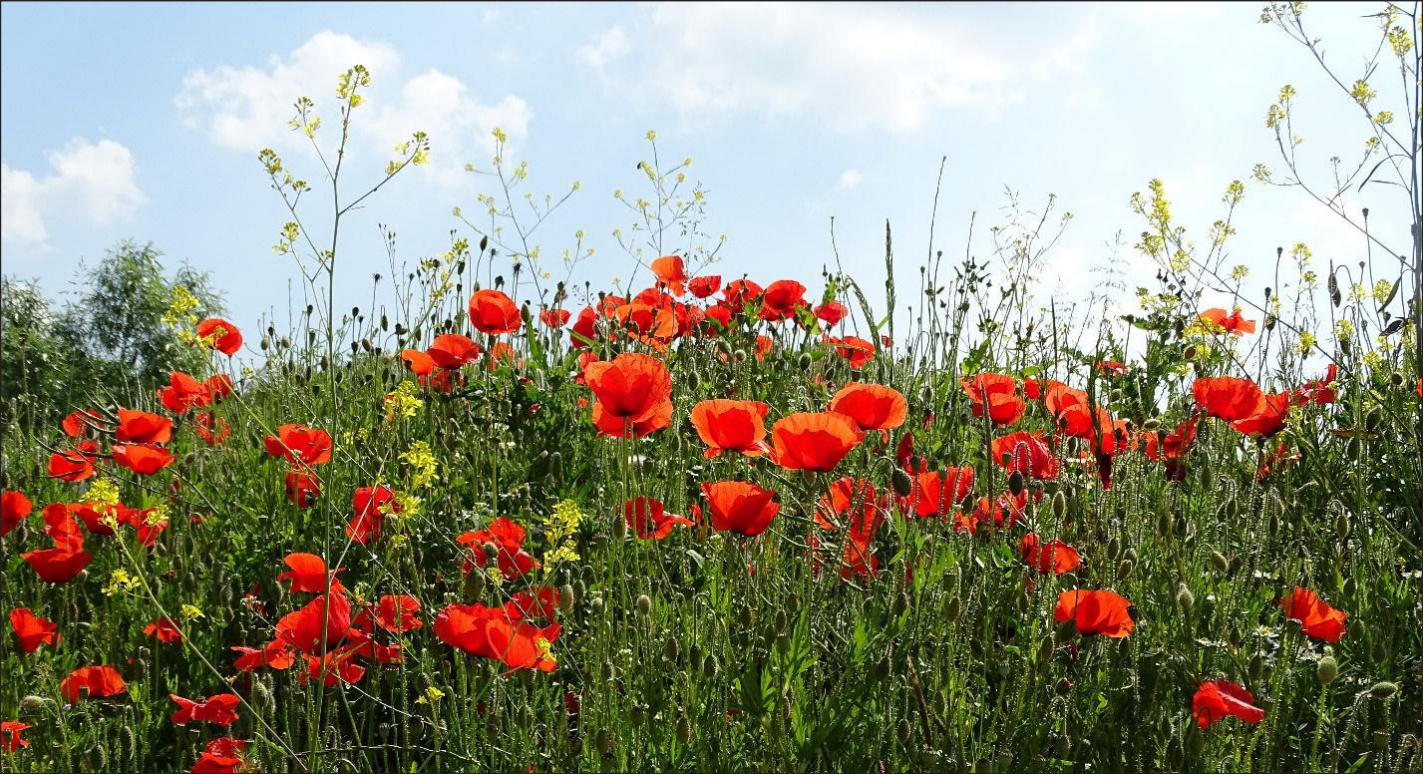
Poppies in Flanders, Belgium. © Ad Meskens/Wikimedia Commons

## Commentary

On May 2, 1915, Canadian Lieutenant Alex Helmer was killed by a shell burst at the Second Battle of Ypres in an area of Belgium known as Flanders. His close friend and brigade surgeon, Major John McCrae, performed his burial rites and later penned the words to his famous poem, “In Flanders Fields,” while sitting on the stoop of an ambulance.^1^ McCrae’s poem would further cement his place in history as a caring physician and talented poet but would also immortalize poppies as symbols of the millions of lives lost during the First World War. His words have outlived him and continue to hold a central place in remembrances of The Great War more than a century later.

Born in Ontario, Canada, on November 30, 1872, John McCrae would begin his military service at an early age, first as a member of the local militia alongside his father and later as an artillery officer during the Boer War in southern Africa. He completed his medical degree at the University of Toronto in 1898 followed by an internship at Johns Hopkins University and fellowship in pathology at McGill University in Montreal. While at Johns Hopkins, McCrae worked alongside Harvey Cushing, the “father of neurosurgery,” and studied under the tutelage of William Osler, a fellow Canadian and one of the cofounders of the Johns Hopkins medical training program.^2^ Osler is still considered one of the great champions of medical education, due in no small part to his encouragement of physicians and trainees to pursue alternate fields of study alongside medicine. He was famous for having a home library to which he would give his students a key, thereby encouraging his pupils to cultivate a penchant for literature while continuing to pursue advances in medical knowledge. One cannot help but wonder at the influence that Osler may have had upon young Doctor McCrae, particularly given the latter’s literary output and later reputation as a polymath—well-traveled, versed in literature, and appreciative of the arts.^3^

The McCrae and Osler families would develop a close connection during this period, as both John and his brother Thomas worked under Osler’s supervision while at Johns Hopkins. These ties would persist in the form of marriage between Thomas McCrae and one of Osler’s nieces but also in more sobering ways: Osler’s son, Edward Revere Osler, would serve alongside John McCrae in France before his death at the Third Battle of Ypres in 1917, becoming yet another poppy in the fields of Flanders.^4^

By all accounts, McCrae excelled in his training at all levels, winning the gold medal while at Toronto, the highest academic accolade for graduating students. He also found success on literary terms, publishing poems in the *Toronto Globe, University Magazine*, and *Saturday Night*, while continuing his medical education. After completing his fellowship in pathology at McGill, McCrae remained in Montreal to establish a clinical practice, solidifying his reputation as a clinician, lecturer, and author. He continued to publish poetry along with medical textbooks and myriad articles in his specialty, ranging from the pathology of burns to an in-depth analysis of scarlet fever.^3^

McCrae was on holiday in England when Germany invaded Belgium on August 4, 1918, leading to declarations of war by Great Britain and Canada. Upon hearing the news, he immediately returned to his home country and volunteered his services as either a combatant or physician, with his preference being the former. He was partially granted his wish when he was assigned to the First Brigade of the Canadian Field Artillery in the dual role of brigade surgeon and second-in-command of the artillery.^5^ His unit arrived in France in February 1915, and he was sent to the Ypres Salient in the Flanders region of Belgium, soon after chlorine gas was first used there by German forces. McCrae established an aid station near the front and remained at that post over the next several months.

Soon after Helmer died in early May, McCrae sat down to pen “In Flanders Fields” while observing the burial sites of his comrades. The poem’s 15 lines are striking for their simplicity and resonance, with poppies given a prominent place in the opening and closing lines. The poem is memorable for the way in which the dead continue to speak to those who are living, a theme McCrae visits in other pieces from his oeuvre, such as “Disarmament” and “The Unconquered Dead.” The poem was an almost immediate success after its publication in *Punch* and would go on to play an important role in military recruiting and victory bond sales throughout the remainder of the war.^6^ In the months following its publication, the Canadian forces realigned their medical corps assets and reassigned McCrae to the No. 3 Canadian General Hospital in Dannes-Camiers, France, where he was later promoted to Lieutenant Colonel. He would remain there on active service, treating soldiers wounded in several major engagements, such as Passchendaele and the Battle of the Somme, until his death from pneumonia and meningitis on January 28, 1918.^5^

McCrae’s untimely death cut short what had already been a brilliant and promising career in the worlds of medicine and literature. Had his life and work continued, he may have joined the ranks of other great literary physicians, such as Chekhov, Williams, and Maugham, or he might have made as lasting an impact upon medicine as Osler or Cushing. Though such ponderings remain unanswered and unanswerable, what is certain is that poppies still blow in Flanders fields, at Passchendaele, the Somme, Verdun, and across countless battlefields throughout the vast fields of France. They, along with lapel pins still worn in parts of Europe and Canada, have been christened by McCrae and pressed into memorial service, urging posterity to remember “the Dead who loved and were loved, but now who lie” in so many early graves of the First World War.

## Disclaimer

The views expressed are solely those of the author and do not reflect the official policy or position of the US Army, US Navy, US Air Force, the Department of Defense, the US Government, or Houston Methodist Hospital.
